# LiDAR–camera fusion for road detection using a recurrent conditional random field model

**DOI:** 10.1038/s41598-022-14438-w

**Published:** 2022-07-05

**Authors:** Lele Wang, Yingping Huang

**Affiliations:** School of Optical-Electrical and Computer Engineering, University of Shanghai for Science & Technology, Shanghai, 200093 China

**Keywords:** Engineering, Mathematics and computing

## Abstract

Reliable road detection is an essential task in autonomous driving systems. Two categories of sensors are commonly used, cameras and light detection and ranging (LiDAR), each of which can provide corresponding supplements. Nevertheless, existing sensor fusion methods do not fully utilize multimodal data. Most of them are dominated by images and take point clouds as a supplement rather than making the best of them, and the correlation between modalities is ignored. This paper proposes a recurrent conditional random field (R-CRF) model to fuse images and point clouds for road detection. The R-CRF model integrates results (information) from modalities in a probabilistic way. Each modality is independently processed with its semantic segmentation network. The probability scores obtained are considered a unary term for individual pixel nodes in a random field, while RGB images and the densified LiDAR images are used as pairwise terms. The energy function is then iteratively optimized by mean-field variational inference, and the labelling results are refined by exploiting fully connected graphs of the RGB image and LiDAR images. Extensive experiments are conducted on the public KITTI-Road dataset, and the proposed method achieves competitive performance.

## Introduction

Road detection is a prerequisite for autonomous driving. Autonomous vehicles are often equipped with multiple sensors for environmental perception, among which LiDAR and cameras are the most informative and commonly used. Road detection has been studied for decades and can be categorized into three types of methods: camera-based, LiDAR-based, and fusion-based methods. Camera-based methods can be performed using images through RGB data^1–3^. LiDAR-based methods segment road areas from 3D point clouds as top-view images^4^, spherical view images^5^, and front view images^6,7^.

Multimodality sensor fusion^8–23^ has been used to improve the perception robustness in autonomous vehicles. A camera provides texture and colours, but the nature of the passive sensor makes it susceptible to variations in environmental lighting. Compared to a camera, LiDAR is not affected by season or illumination conditions and offers 3D geometry information to complement visual data shortcomings. Fusion-based methods are thought to overcome the weakness of each sensor case and exhibit promising performance. Semantic segmentation is an efficient style used to analyze the sensor inputs for autonomous driving, and the image and point cloud are classified into semantic classes. Conditional random field (CRF) is an effective tool used to integrate the results obtained from each sensor and therefore refines the segmentation result. CRF^24^ is a discriminative probability model. Since pixel labels can be regarded as random variables, CRF can be used to model the labelling problem. Normally, CRF is defined as an undirected graph with pixels as nodes. It can be solved by an approximate graph inference algorithm by minimizing the energy function. The function contains unary and pairwise potentials. The unary potential is only concerned with the node itself and determines the probability of the node being labelled. The pairwise potential describes interactions between neighbouring nodes and is defined as similarity.

Existing works for road detection using CRF^17–23^ have the following issues. (1) These works do not make full use of results (information) from two sensors. Regarding the energy function, method^17^ only uses the result generated by RGB images as the input of the unary term. Other works^18,19,22^ use both results to define the unary term, but the result generated by the point cloud takes effect only as a supplement because point cloud data are sparse. In the pairwise term of the energy, most works^18,20,22^ only use interactions between neighbouring nodes of the image and do not consider the point cloud information at all. In summary, the correlation between the two modalities of data is ignored. (2) Existing works use the graph cut-based algorithm to conduct graph inference. However, graph cut-based inference is only applicable in a locally connected graph that only considers the local interaction. Ideally, the undirected graph should be a fully connected graph that considers the local and global interactions of the RGB image or LiDAR image.

To address the issues mentioned above, the *recurrent conditional random field (R-CRF)* model is proposed, which employs mean-field variational inference to conduct graph inference rather than a graph cut-based algorithm. Formulated as a recurrent model, mean-field variational inference performs iterative optimization through a series of message-passing steps, and each step updates one variable by aggregating information from all other variables. Because the pairwise potential can be considered a compounding of linear combinations of Gaussian kernels, the message-passing step in mean-field variational inference can be considered a convolution. R-CRF using mean-field variational inference dramatically reduces the computational complexity, therefore enabling us to conduct graph inference in the form of a fully connected graph. On the other hand, the proposed R-CRF model makes full use of the results (information) of two sensors. It takes probability scores generated by two modalities of data as the unary potential term, and both the RGB image and the densified LiDAR images are utilized as pairwise potential terms to encode the contextual consistency. Followed by such a fusion process, the proposed model possesses a considerable error correction capability.

Compared to the literature, the major contributions are as follows:

1. The R-CRF model is proposed to fully integrate the results (information) of multisensor data (images and point clouds) in a probabilistic way. Specifically, the densified LiDAR image and RGB image are reasonably added to the pairwise input to encode the contextual consistency.
2. Mean-field variational inference is utilized to solve the graph inference problem rather than graph cut-based inference; therefore, the labelled results can be refined through a fully connected graph that uses the local and global interaction of the RGB image or LiDAR image. Specifically, the message-passing step in inference is reformulated to a convolution with a truncated Gaussian kernel.
3. We conduct extensive experiments on the KITTI road benchmark, and the results indicate that the approach in this paper is robust to the environment and achieves promising detection performance.

## Related work

Various approaches have been developed and can be divided into two groups in terms of the use of sensors: one-sensor-based and multiple-sensor fusion-based methods.

### One-sensor-based road detection

Departing from fully convolutional networks (FCNs), diverse structures have been proposed to provide accurate pixelwise prediction results for the task of road detection. MultiNet^1^ was proposed through a unified architecture for multiple tasks. An encoder and decoder scheme named RBNet^2^ was applied to recollect features at different scales. Additional driving scene images were generated by *Fan*^3^. However, the quality of the image is heavily impacted by weather conditions, reducing the accuracy.

Other related approaches focus on using point clouds, which utilize the geometric properties measured from sparse range data. Compared with those in diverse images, geometric characteristics in LiDAR are relatively simple and easier to learn. *Fernandes*^6^ obtained an accurate road estimation result through the sliding window technique and utilized morphological processing to classify roads from point clouds. The projection-based method^25,26^ projected point clouds into the BEV view or spherical front view. These representations are adequate for real-time systems. LoDNN^4^ transformed unstructured LiDAR data into a top-view representation by basic statistics, such as the point number, mean, average, standard deviation, minimum and maximum, and then those maps were employed as input for a CNN to achieve the desired result. *Lyu*^5^ arranged the points into specific views as input, and then, the proposed FCN was implemented on an FPGA. *Gu*^7^ obtained an inverse map and acquired the approximate road regions by extracting the vertical and horizontal histograms.

### Multiple sensor fusion-based road detection

For robust environment perception in autonomous vehicles, to eliminate inherent disadvantages and absorb the essence of various sensors, data-fusion approaches for road detection can be classified into the following three levels:

1. Early level fusion: Different types of sensor data are combined to produce a new kind of data through data alignment, preserving all information. *Wulff*^8^ proposed the UGrid-Fused approach, a multidimensional occupation grid representation based on BEV, which can be imported into the FCN. Each cell in UGrid-Fused contains 15 statistics, including a binary map, a count map, an obstacle map, six height measurement maps and six reflectivity intensity maps. *Yu*^9^ transformed the bird’s eye view of two modes to facilitate data transfuser. Lee and Park^10^ focused on the idea of contracting the size of inputs and expanding the perceptual field of the network. The two modalities are transformed into spherical coordinates, and the height data of the point cloud and R, G, and B channels are superimposed on channels and then subsequently fed into the modified SegNet network.
2. Middle level fusion: Features from multiple sensor data are used to accompany the scenario. *Chen*^11^ solved the feature space mismatch problem by performing altitude difference on LiDAR data and then a cascaded fusion structure was implemented based on the DCNN. *Caltagirone*^12^ used RGB image data and the interpolated 2D LiDAR image data by *Premebida*^13^ into a modified CNN. These fusion strategies in deep networks are essentially an addition/concatenation operation.
3. Late level fusion: The data are operated individually by each of their networks, and then, the 2D and 3D results are integrated based on mutual relationships or probabilistic modelling. RES3D-Velo^14^ projected point clouds to image space and constructed a graph by Delaunay triangulation, applying spatial relationships to discriminate obstacles. Since it only uses a cross-calibration parameter to obtain points, the colour information is not utilized at all. After projecting points to the image, *Xiao*^15^ employed plane estimation to identify points on the ground plane. The Gaussian model was used to learn image features, and pixels were also classified through this model. However, this segmentation process is implemented only on images, which has a substantial limitation. *Jihun Park*^16^ proposed drivable region identification for dirt roads by fusing semantic segmentations of modalities. The two segmentation results are integrated into the BEV grid.

Current popular CRF-based^24^ methods were proposed for road detection. Fusion with CRF^17^ was performed at the unary stage, and CRF was only used as postprocessing for superpixel labelling. FusedCRF^18^ utilized boosting classifiers for two modalities, and the result of the LiDAR classifier was only available as an additional observation. The pairwise term only considered the image difference between adjacent pixels. A hybrid model^19^, an advanced CRF fusion model, further considered the interactions between 3D points, image pixels, and one between them. The results were optimized with sub-CRFs. The features of each sensor were traditionally extracted. Due to the sparsity of LiDAR data, the imbalance still existed. *Gu*^20^ proposed a modified convolutional network (IDA-FCN) on RGB images and a line-scanning strategy on point clouds. Late fusion was performed, and the LiDAR result still worked as a supplement as in FusedCRF^18^. The depth images generated by joint bilateral filters^21^ and features of both modalities were extracted and input into the Adaboost classifier for a coarse result. The fine results were obtained by the CRF operation. *Gu* also^22^ applied a fast height-difference-based approach to generate dense results in a spherical view to blend the outputs of the two modalities in a balanced way. The energy contained the 2D unary potential, 3D potential, and 2D–3D pairwise potential. Reference^22^ further considered the distribution of projection points and proposed an improved Delaunay triangular upsampling strategy^23^.

## Method

**Figure 1:**
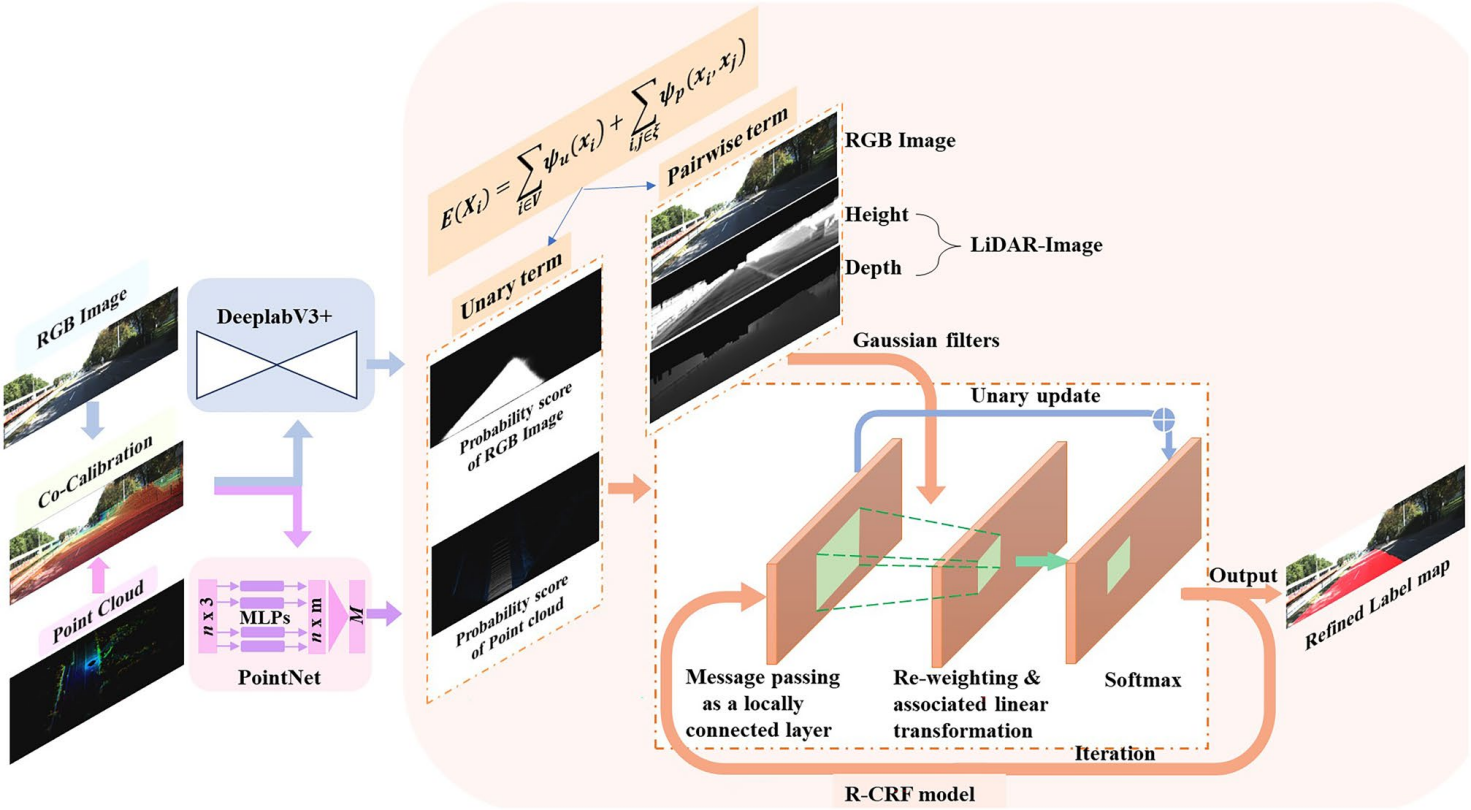
Method framework in this paper. The output of the image space can be seen as the terminal result for the test.

The architecture is shown in Fig. 1. Both modalities are aligned through cross-calibration, and corresponding depth and height images are generated. The generated LiDAR maps are integrated into pairwise potentials in the R-CRF model as described below. The RGB image is input into the DeepLab V3 + semantic segmentation network, while the 3D point clouds are input into the PointNet segmentation network. The segmentation results generated from the two networks are probability scores for pixels. The proposed recurrent conditional random field model is then followed to integrate the results (information) of two modalities of data. Specifically, the R-CRF model takes segmentation results as a unary term. Meanwhile, it adds the RGB image, densified LiDAR depth and height images as pairwise terms to make the proposed approach more robust. Finally, the proposed method is iteratively optimized by mean-field variational inference.

### Data preprocessing

LiDAR scans the surrounding environment and obtains a large number of point clouds. To extract a meaningful point cloud that corresponds to pixels, it is necessary to preprocess the data and remove the redundant points.

#### Data alignment

Both point cloud and RGB images are organized by the different data structures and have different coordinate systems. LiDAR data consist of numerous points in the real world, and each LiDAR point is identified by a 3D coordinate vector. The RGB image consists of pixels, and each pixel is described by an RGB value. In this section, the alignment is introduced. Point ${P}_{lidar}=({x}_{l},{y}_{l},{z}_{l},1{)}^{T}$ in the 3D LiDAR coordinate system is transformed into 3D point ${P}_{cam}=({x}_{c},{y}_{c},{z}_{c},1{)}^{T}$ in camera coordinates. The 3D points in the camera coordinate system ${P}_{cam}$ with ${z}_{c}>0$ (front view of the camera) are turned into ${p}_{cam}=({u}_{c},{v}_{c},1{)}^{T}$ in image coordinates. The transformation equation is as follows:

1 $$\begin{array}{*{20}c} {P_{cam} = R_{rect}^{0} \cdot T_{velo}^{cam} \cdot P_{lidar} } \\ \end{array}$$

2 $$\begin{array}{*{20}c} {p_{cam} = T_{proj } \cdot P_{cam} } \\ \end{array}$$

3 $$\begin{array}{*{20}c} {T_{velo}^{cam} = \left[ {\begin{array}{*{20}c} {R_{velo}^{cam} } & {t_{velo}^{cam} } \\ 0 & 1 \\ \end{array} } \right]} \\ \end{array}$$

where ${R}_{rect}^{0}$ is the rotation matrix, ${T}_{velo}^{cam}$ is the transformation matrix, and ${T}_{proj}$ is the projection matrix.

**Figure 2:**
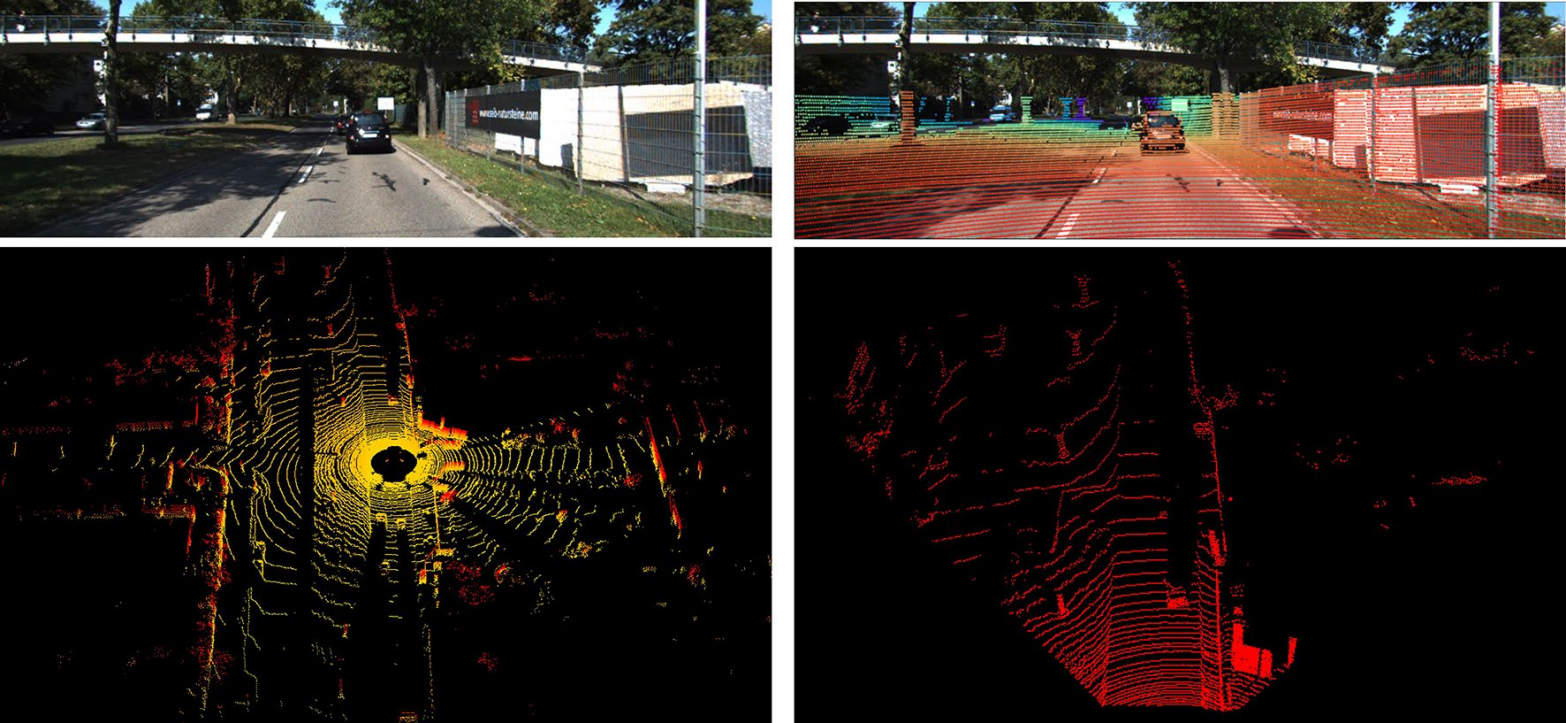
Illustration of the image and the corresponding point cloud alignment. The top left is an RGB image from a camera with 1242 × 375 pixels. The bottom left is the LiDAR point cloud with 64 channels in the 3D real world. The bottom right shows the point cloud (only the data overlapping the front view are displayed). The top right shows the demo of the point cloud and RGB image alignment, where the colour indicates the distance (red dots are near and blue dots are far).

The above transformation is applied to each point. Note that points with positive Z-values remain. Figure 2 shows the data alignment, including the image (top left), data alignment (top right), a point cloud generated by a LiDAR scanner in the 3D real world coloured by height (bottom left), and LiDAR (FOV of the image, bottom right).

#### Dense LiDAR-image map generation

**Figure 3:**
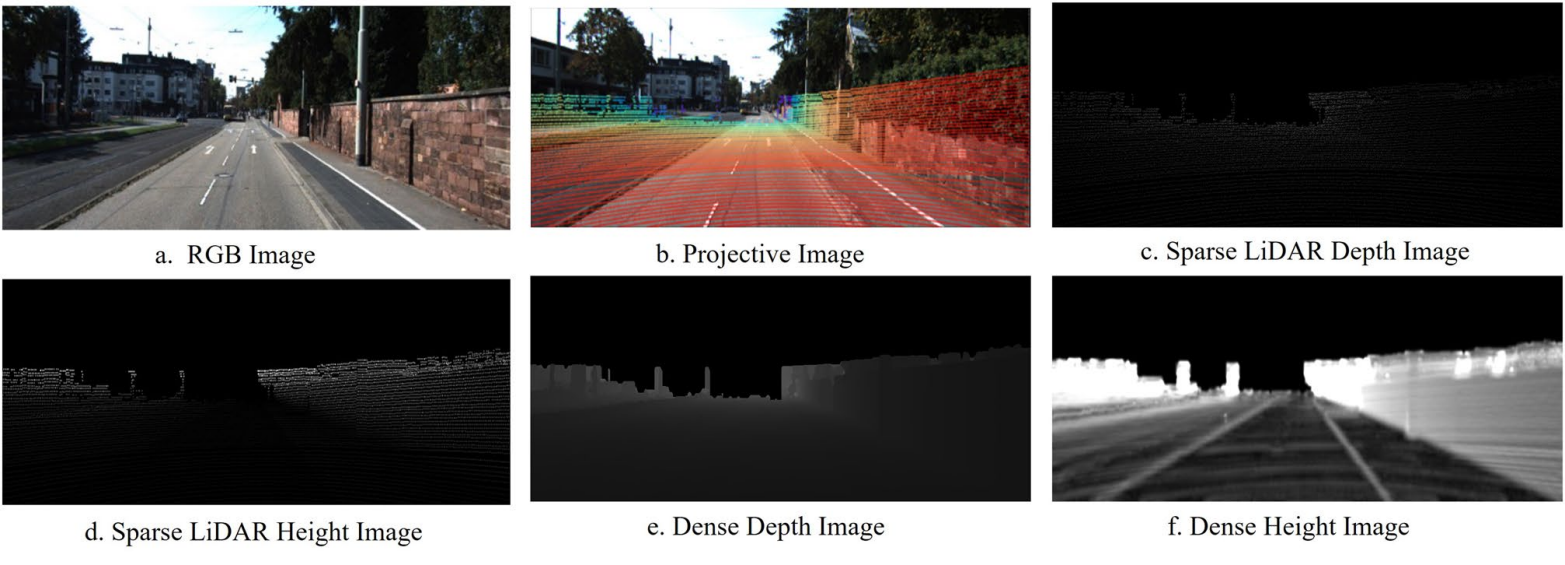
Dense LiDAR image generation: (**a**) RGB image, (**b**) projective image (RGB image with a superimposed sparse point cloud), (**c**) generated sparse LiDAR depth image, (**d**) generated sparse LiDAR height image, (**e**) generated dense LiDAR depth image, and (**f**) generated dense LiDAR height image.

After transformation, three-channel tensors with the same dimension are received. Each channel encodes 3D spatial coordinates. Due to the sparse nature of LiDAR data, projected points with corresponding planes are much sparser than the associated image; thus, the sparse LiDAR image representation is processed to generate the dense representation. As shown in Figure 3, we utilize the strategy^13^ to obtain a dense depth image, as shown in Fig. 3e, and the height transformation operation^11^ to obtain a height difference image, as illustrated in Fig. 3f, which can better preserve the characteristics. In Fig. 3e, pixel values become larger or brighter with increasing distance. While road and nonroad areas can be similar, height maps are very helpful in distinguishing road areas, as roads are usually lower in height than on roads.

#### LiDAR sample labelling

**Figure 4:**
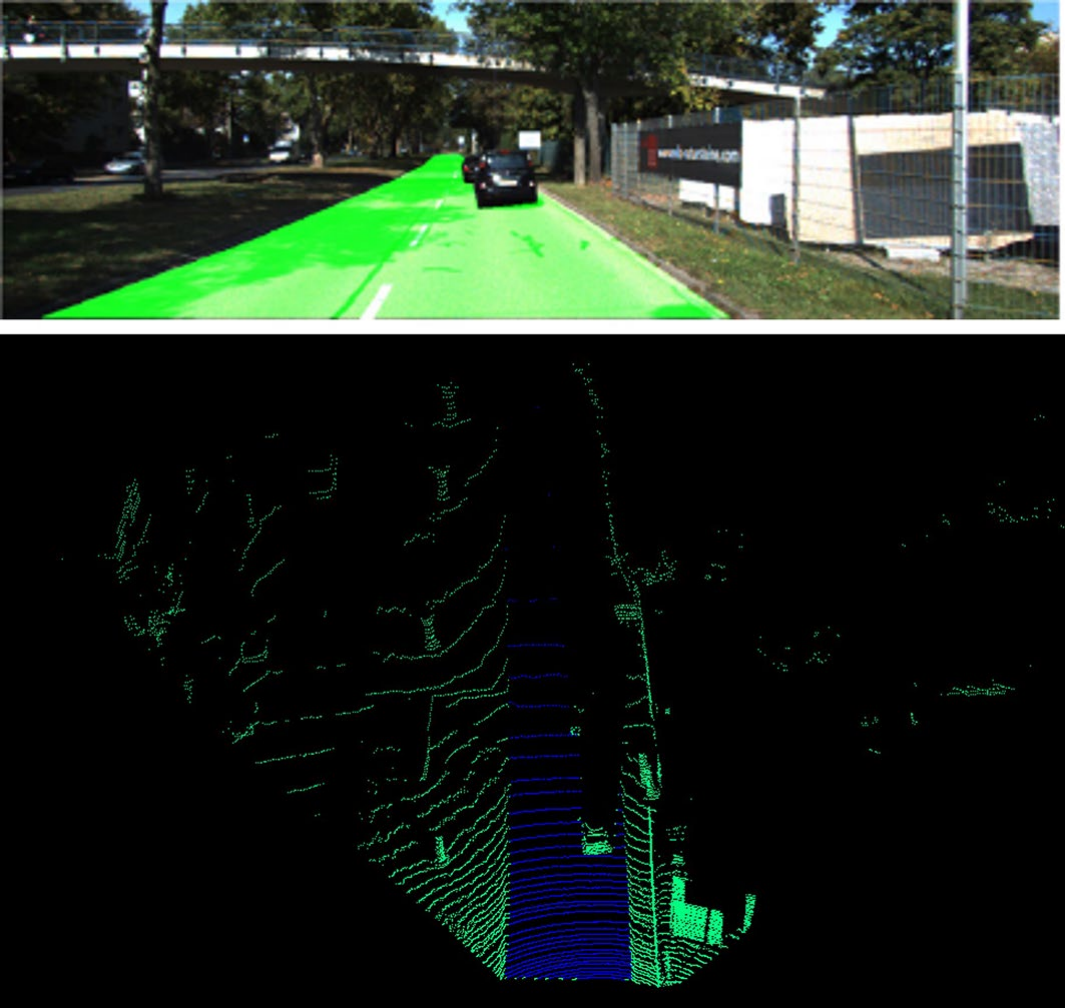
Illustration of labelling.

The labelling of point clouds is extremely labour intensive. Because modalities are already aligned, the label of the corresponding point cloud can be easily obtained from the ground truth image. The equation is presented as follows:

4 $$\begin{array}{*{20}c} {Lable_{LiDAR}^{i} = \left\{ {\begin{array}{*{20}c} {1,} & {if \quad Label_{Image}^{{T_{LtoI} \quad\times LiDAR^{i} }} = road \, area} \\ {0,} & {otherwise} \\ \end{array} } \right\}} \\ \end{array}$$

where ${Lable}_{LiDAR}^{i}$ indicates the label of the $ith$ point cloud. In addition, ${Label}_{Image}^{{T}_{LtoI} \quad \times LiDAR^{i}}=road \, area$ means that the semantic label $\left({Label}_{Image}\right)$ of the projected image pixel of the $ith$ point cloud (${T}_{LtoI}\times {LiDAR}^{i}$) is a road. Figure 4 illustrates the labelling results.

#### RGB image and point cloud detection

To better use both modalities, before applying the R-CRF model, each modality should be independently trained through types of semantic segmentation algorithms to identify road areas. PointNet^27^ is a pioneer in consuming 3D point clouds. It directly models disordered point sets and captures local and global point features via MLP layers. After data alignment, point clouds within the image’s field are extracted, and the processed point clouds are analyzed through the 3D segmentation network PointNet^27^, which categorizes point clouds into two classes: road points and other points. DeeplabV3+^28^ is an existing competitive image semantic segmentation method. It exploits the encoder–decoder structure to connect different-level features at different scales. It classifies all pixels into semantic classes. To accelerate the inference process, a lightweight network MobileNet-V2 is used as the backbone. Through two types of semantic segmentation networks, probabilistic scores can be obtained.

### Recurrent conditional random field

#### General CRF-based labelling in computer vision

The conditional random field (CRF) model is a probabilistic graphical model that models a probability distribution of pixel labels and is conditioned on global observations. Consider a random field $X={\{X}_{1},{X}_{2},\dots {X}_{N}\}$ defined as the random variables to be inferred from RGB image $Y$. Every random variable ${X}_{i}$ takes a label from $\mathcal{L}=\left\{{l}_{1},{l}_{2},\dots {l}_{k}\right\},$ where $k$ is the semantic label. Any possible assignment of all random variables is called labelling, which can take values from $\mathcal{L}$.

The general CRF-based labelling model is defined over an indirect graph $G=\left(V,\xi \right)$, where $V$ contains all pixels, $V={X}_{1},{X}_{2},\dots {X}_{N}$, N is the size of the RGB image, and $\xi$ defines the connectivity between random variables. For each pixel, the neighbourhood system usually adopts 4 or 8 connections. The general energy function is as follows:

5 $$\begin{array}{*{20}c} {P\left( {X = x{|}Y} \right) = \frac{1}{Z\left( Y \right)}\exp \left( { - E\left( {X|Y} \right)} \right),and\quad Z\left( Y \right) = \mathop \sum \limits_{x} \exp \left( { - E\left( {X|Y} \right)} \right)} \\ \end{array}$$

where $Z$ is the partition function. The Gibbs energy function can be written as follows:

6 $$\begin{array}{*{20}c} {E\left( {X|Y} \right) = \mathop \sum \limits_{i \in V} \psi_{u} (x_{i} ) + \mathop \sum \limits_{i,j \in \xi } \psi_{p} (x_{i} ,x_{j} )} \\ \end{array}$$

For notational convenience, it is wise to omit the conditioning on Y, and ${\psi }_{u}\left(\bullet \right)$ is the unary potential, the cost of assigning label ${x}_{i}$ to pixel $i$. ${\psi }_{p}\left(\bullet \right)$ is the pairwise potential, the cost of assigning labels ${x}_{i}$ and ${x}_{j}$ to pixels $i$ and $j$.

#### R-CRF model

The traditional CRF model generally considers the result of the RGB image as a unary term, and it only requires connecting 4 or 8 local neighbours in the pairwise potential. The graph inference is based on Graph-cut. This approach leads to inefficient local optimization of the CRF model and cannot capture global features. Therefore, the proposed R-CRF model makes full use of the results (information) of two sensors. Each modality is independently processed with its own semantic segmentation network. It takes probability scores generated by two modalities of data as the unary potential term, and both RGB images and densified LiDAR images are utilized as pairwise potential terms to encode the contextual consistency. Then, the energy function is iteratively optimized by mean-field variational inference, and the labelling results are refined through a fully connected graph that uses the local and global interaction of the RGB image and LiDAR image. The R-CRF model can be formulated by minimizing the energy function defined as follows: $x$ denotes the labels assigned to pixels.

7 $$\begin{array}{*{20}c} {min_{x} E\left( {X = x} \right) = \mathop \sum \limits_{i \in V} \psi_{u} (x_{i} ) + \mathop \sum \limits_{{\begin{array}{*{20}c} {i < j} \\ {i,j \in V} \\ \end{array} }} \psi_{p} (x_{i} ,x_{j} )} \\ \end{array}$$

#### Unary potential

${\psi }_{u}{(x}_{i})$ can be regarded as the prior distribution. It takes the negative log-likelihood of variable $X$ predicted by the outputs of the segmentation network.

8 $$\psi_{u} (x_{i} ) = \psi_{u}^{I} (x_{i} ) + \psi_{u}^{L} (x_{i} )$$

9 $$\psi_{u}^{I} (x_{i} ) = - log\left( {H_{P}^{I} (x_{i} )} \right),\quad \psi_{u}^{L} (x_{i} ) = - \lambda \log \left( {H_{P}^{L} \left( {x_{i} } \right)} \right){ }$$

${\psi }_{u}^{L}{(x}_{i}$) and ${\psi }_{u}^{I}{(x}_{i})$ represent the potentials of the point cloud and image data, respectively. ${H}_{P}^{I}\left({x}_{i}\right)$ and ${H}_{P}^{L}\left({x}_{i}\right)$ are the results of each modality segmentation network. $\lambda$ is utilized to balance the tradeoff between the terms in (9). For equal fusion, $\lambda$ is set to 1 in the experiment.

#### Pairwise potential

${\psi }_{p}{(x}_{i},{x}_{j})$ consists of a weighted sum of Gaussian functions and is only related to the difference between pixels $i$ and $j$. It encourages neighbouring pixels to have the same labels and has a smoothing effect on the labelling result. ${\psi }_{u}{(x}_{i})$ can be regarded as the prior distribution. It takes the negative log-likelihood of variable $X$ predicted by the outputs of the segmentation network.

10 $$\psi_{p} (x_{i} ,x_{j} ) = u\left( {x_{i} ,x_{j} } \right)\mathop \sum \limits_{m = 1}^{M} w^{m} k^{\left( m \right)} \left( {f_{i} ,f_{j} } \right) = u\left( {x_{i} ,x_{j} } \right)g\left( {f_{i} ,f_{j} } \right)$$

where ${k}^{\left(m\right)}$ for m = 1…, M is the Gaussian kernel applied on feature vectors $f$ and ${w}^{m}$ is the corresponding coefficient.

The label compatibility function $u\left({x}_{i},{x}_{j}\right)=1$ if ${ x}_{i}\ne {x}_{j}$ and is 0 otherwise showing the compatibility between different label pairs. Traditional methods only utilize features extracted from the RGB modality, whereas in this paper, several Gaussian kernels consider point clouds along with RGB images.

The first Gaussian kernel in Eq. (11) is observed by the RGB image; the former is called the Gaussian *appearance kernel,* which boosts nearby pixels with similar colours that may belong to the same class. The latter is a Gaussian *spatial kernel* called a smoothing kernel; it removes minor obscure regions in the same way that previous models do.

11 $$\begin{array}{*{20}c} {g^{\left( 1 \right)} \left( {f_{i} ,f_{j} } \right) = w^{\left( 1 \right)} \exp \left( { - \frac{{\left| {p_{i} - p_{j} } \right|^{2} }}{{2\theta_{\alpha }^{2} }} - \frac{{\left| {I_{i} - I_{j} } \right|^{2} }}{{2\theta_{\beta }^{2} }}} \right) + w^{\left( 2 \right)} \exp \left( { - \frac{{\left| {p_{i} - p_{j} } \right|^{2} }}{{2\theta_{\gamma }^{2} }}} \right)} \\ \end{array}$$

where ${p}_{i}$ and ${p}_{j}$ are the positions in image coordinates, ${I}_{i}$ and ${I}_{j}$ are colour values, and ${\theta }_{\alpha }$, ${\theta }_{\beta }$, and ${\theta }_{\gamma }$ are kernel parameters.

The second and third Gaussian kernels are observed by the point cloud, and the height and depth maps are obtained from the aligned point cloud. The second Gaussian kernel is *a height bilateral kernel*:

12 $$\begin{array}{*{20}c} {g^{\left( 2 \right)} \left( {f_{i} ,f_{j} } \right) = w^{\left( 3 \right)} \exp \left( { - \frac{{\left| {p_{i} - p_{j} } \right|^{2} }}{{2\theta_{\varepsilon }^{2} }} - \frac{{\left| {H_{i} - H_{j} } \right|^{2} }}{{2\theta_{\eta }^{2} }}} \right)} \\ \end{array}$$

where ${H}_{i}$ and ${H}_{j}$ are height values and ${\theta }_{\varepsilon }$ and ${\theta }_{\eta }$ are kernel parameters. The third kernel is the *distance bilateral kernel*, which assumes that nearby pixels with close distances are likely to be the same semantic:

13 $$\begin{array}{*{20}c} {g^{\left( 3 \right)} \left( {f_{i} ,f_{j} } \right) = w^{\left( 4 \right)} \exp \left( { - \frac{{\left| {p_{i} - p_{j} } \right|^{2} }}{{2\theta_{\sigma }^{2} }} - \frac{{\left| {D_{i} - D_{j} } \right|^{2} }}{{2\theta_{\omega }^{2} }}} \right)} \\ \end{array}$$

where ${D}_{i}$ and ${D}_{j}$ are the values of the distance in the LiDAR coordinates. ${\theta }_{\sigma }$ and ${\theta }_{\omega }$ are kernel parameters. The parameters ${\theta }_{\alpha }$, ${\theta }_{\beta }$, ${\theta }_{\gamma }$, ${\theta }_{\varepsilon }$, ${\theta }_{\eta }$,${\theta }_{\sigma }$ and ${\theta }_{\omega }$ control the scale of the Gaussian kernel.

#### Mean field iteration in the recurrent-CRF model

Minimizing Eq. (6) yields the most likely label assignment for the given data. Extract minimization of the equation is intractable, so the mean-field variable inference algorithm^29–34^ is proposed to approximately and efficiently solve the fully connected graph. Inspired by the work of ConvCrf^31^, we bring the conditional independence assumption to the fully connected CRF model, and the message-passing step is reformulated to a convolution with a truncated Gaussian kernel. Following^30,31^, we approximate the Gibbs distribution ${\varvec{P}}\left({\varvec{X}}\right)$ with the mean-field distribution ${\varvec{Q}}\left({\varvec{X}}\right)$ to minimize the KL divergence between ${\varvec{P}}\left({\varvec{X}}\right)$ and ${\varvec{Q}}\left({\varvec{X}}\right)$. The form of $Q\left( X \right)$ is as follows:

14 $$\begin{array}{*{20}c} {Q\left( {\text{X}} \right) = \mathop \prod \limits_{{{\text{i}} = 1}}^{{\text{N}}} {\text{Q}}_{{\text{i}}} \left( {{\text{X}}_{{\text{i}}} } \right)} \\ \end{array}$$

Mean-field variational inference is usually implemented by continuously updating the distribution $Q\left( X \right)$ iteratively, and finally, the optimal solution is obtained, which is expressed as follows:

15 $$\begin{aligned} KL(Q\left( X \right)||P(X|Y)) & = \mathop \sum \limits_{X} Q\left( X \right)log\frac{Q\left( X \right)}{{P\left( {X|Y} \right)}} \\ & = \mathop \sum \limits_{X} Q\left( X \right)log\frac{Q\left( X \right)Z\left( Y \right)}{{\exp \left( { - E\left( {X,Y} \right)} \right) }} \\ & = \mathop \sum \limits_{X} Q\left( X \right)E\left( {X,Y} \right) + \mathop \sum \limits_{X} Q\left( X \right)logQ\left( X \right) + logZ\left( Y \right) \\ \end{aligned}$$

The iterative update equation is as follows:

16 $$Q_{i} \left( {x_{i} = l} \right) = \frac{1}{{Z_{i} }}\exp \left\{ { - \psi_{u} \left( {x_{i} } \right) - \mathop \sum \limits_{{l^{\prime} \in L}} u\left( {l,l^{\prime}} \right)\mathop \sum \limits_{m = 1}^{k} w^{\left( m \right)} \mathop \sum \limits_{i \ne j}^{k} k^{\left( m \right)} \left( {f_{i} ,f_{j} } \right)Q_{j} \left( {l^{\prime}} \right)} \right\}$$

A brief description of how to break the update equation down into simpler steps in Algorithm 1 is provided. It is composed of six steps:
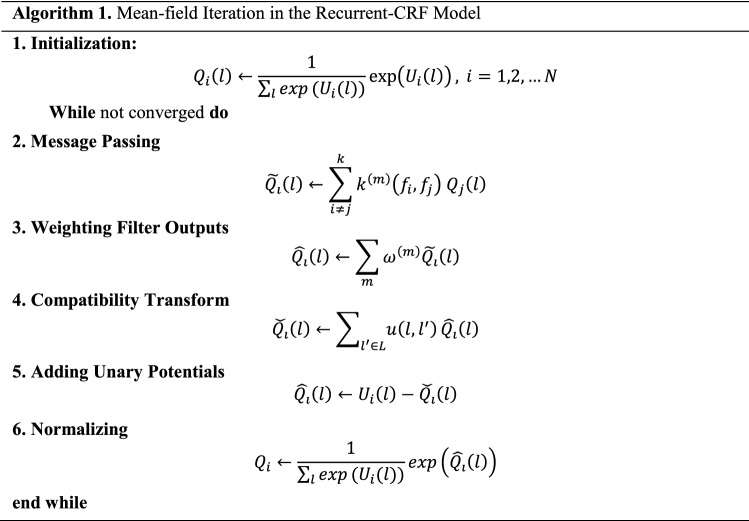


**Step 1: Initialization.** The probability scores obtained from segmentation algorithms are utilized for initialization.

**Step 2: Message Passing.** The message-passing step involves filtering the approximated marginal. Gaussian kernels based on images are processed to obtain differences in Eqs. (11) to (13). As the distance between two pixels increases, the value of the Gaussian kernels mentioned above decreases very quickly. Therefore, assuming that the label distribution of two data $i \,\,\mathrm{and}\,j$ are conditionally independent, for all pixels whose Manhattan distance $d\left(i,j\right)>k$, where $k$ is a hyperparameter, the pairwise potential is zero, greatly reducing the complexity of the pairwise potential. This reflects the correlation between a pixel and others.

**Step 3: Weighting Filter Outputs.** We apply Gaussian kernels to filter the probability map in step 2; this step can be seen as a $1\times 1$ convolution.

**Step 4: Compatibility Transform.** This step is utilized to determine the extent of how it changes the distribution. This step can be seen as convolution with the $1\times 1$ kernel.

**Step 5: Adding Unary Potentials.** We update it by adding the unary potential received from step 1 to the result of step 4.

**Step 6: Normalization.** SoftMax is used for normalization.

The output of this module is a refined probability map that can be further refined by iterative applications.

**Figure 5:**
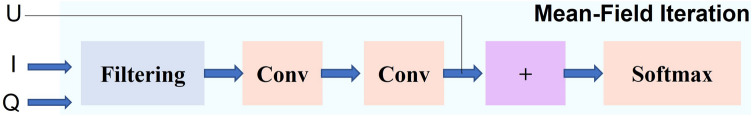
Updated equation for the mean-field inference of^36^ decomposed into smaller steps, where one iteration can be seen as a neural network.

Generally, one iteration can be modelled as a bunch of ordinary CNN layers. By processing multiple iterations, the output of one iteration becomes the input for the next iteration, as illustrated in Fig. 5.

## Experiments

### Dataset and metrics

The R-CRF model is evaluated on the broadly utilized KITTI ROAD benchmark^35^. The ROAD dataset includes corresponding calibration parameters, ground-truth images, RGB images, point clouds, and scripts for evaluation. It consists of 289 labelled frames for the training set and 290 frames for the testing set. Terminal results are evaluated on KITTI’s online server. For road detection, the KITTI dataset presents four scenarios: urban unmarked road (UU), urban marked road (UM), urban multiple marked lanes (UMM) and all three urban subsets (URBAN). In addition, a category called URBAN is calculated, which supplies an overall score. In this case, only the road area is considered, and the lane detection task is ignored.

Following benchmark evaluation, the KITTI-ROAD dataset provides the maximum F-measure at the pixel level in bird’s eye view (BEV) space. Principal metric matrix values are used to evaluate the accuracy, including MaxF (maximum F1), PRE (precision), REC (recall), AP (average precision), FPR (false-positive rate) and FNR (false-negative rate). The definition of the matrix is as follows:

17 $$Precision = \frac{TP}{{TP + FP}},\quad Recall = \frac{TP}{{TP + FN}}$$

18 $$\begin{array}{*{20}c} { AP = \frac{TP + TN}{{TP + FP + TN + FN}}} \\ \end{array}$$

19 $$\begin{array}{*{20}c} { FPR = \frac{FP}{{FP + TN}}, \quad FNR = \frac{FN}{{TP + FN}}} \\ \end{array}$$

20 $$MaxF = {\text{max}}(\frac{{2 \times { }Precision \times Recall}}{Precision + Recall}) = \frac{2TP}{{2TP + FP + FN}}$$

where $TP$, $FP$, $TN$, and $FN$ represent the number of samples. Precision and recall bring different insights into the method’s performance: low precision implies that many background pixels are sorted as roads, while low recall indicates that road surfaces are not detected. The KITTI benchmark ranks all methods according to $\mathrm{Max}F$.

### Implementation details

A modified Deeplabv3 + network is used for the 2D segmentation network, and PointNet is used for the 3D segmentation network. For a more focused view, the input of the RGB camera is resized to $1242\times 375$, and the learning rate for image training is set to 0.001. The input of LiDAR point clouds is rectified; for one image, approximately 20,000 points are used in the camera field of view, and the learning rate is set to 0.001 for point cloud segmentation. Furthermore, the number of epochs and batch size are set to 400 and 4, respectively. The parameters of the R-CRF model include $\lambda$, ${w}^{\left(1\right)}$, ${w}^{\left(2\right)}$, ${w}^{\left(3\right)}$, and ${w}^{\left(4\right)}$, and ${\theta }_{\alpha }$, ${\theta }_{\beta }$, ${\theta }_{\gamma }$, ${\theta }_{\varepsilon }$, ${\theta }_{\eta }$,${\theta }_{\sigma }$, and ${\theta }_{\omega }$ are set empirically. Specifically, $\lambda$ is set to 1; ${w}^{\left(1\right)}$, ${w}^{\left(2\right)}$, ${w}^{\left(3\right)}$, and ${w}^{\left(4\right)}$ are set to 100, 80, 80, and 100, respectively; and ${\theta }_{\alpha }$, ${\theta }_{\beta }$, ${\theta }_{\gamma }$, ${\theta }_{\varepsilon }$, ${\theta }_{\eta }$,${\theta }_{\sigma }$, and ${\theta }_{\omega }$ are set to 10, 10, 1, 10, 10, 10, and 10, respectively. The proposed framework is implemented on an Ubuntu 18.04 operating system, and the environment is carried out with an NVIDIA 1080 TI GPU.

### Experimental results

#### Ablation study

We compare the results obtained from image only, point cloud only and the proposed fusion method. The experiments are conducted on the validation dataset. The results are illustrated in Table 1. Image only means that only the image-segmentation algorithm is employed, and point cloud only means that only the point cloud segmentation algorithm is employed. The method one is the whole framework described in this paper, with the input of multimodality data. The fusion model achieves the best performance, with a MaxF score of 94.64%, an improvement of 2.76% over that of the image-based method and 1.26% over that of the point cloud-based method. The fusion model achieves a significant improvement through a combination of geometric properties and colour information.

**Table 1:** Comparison results under different modalities on validation dataset (/%).

Modality	MaxF	AP	PRE	REC
Image only	91.88	88.12	92.45	91.21
Point cloud only	93.38	92.71	94.32	92.73
Fusion	94.64	93.23	95.06	94.22

**Figure 6:**
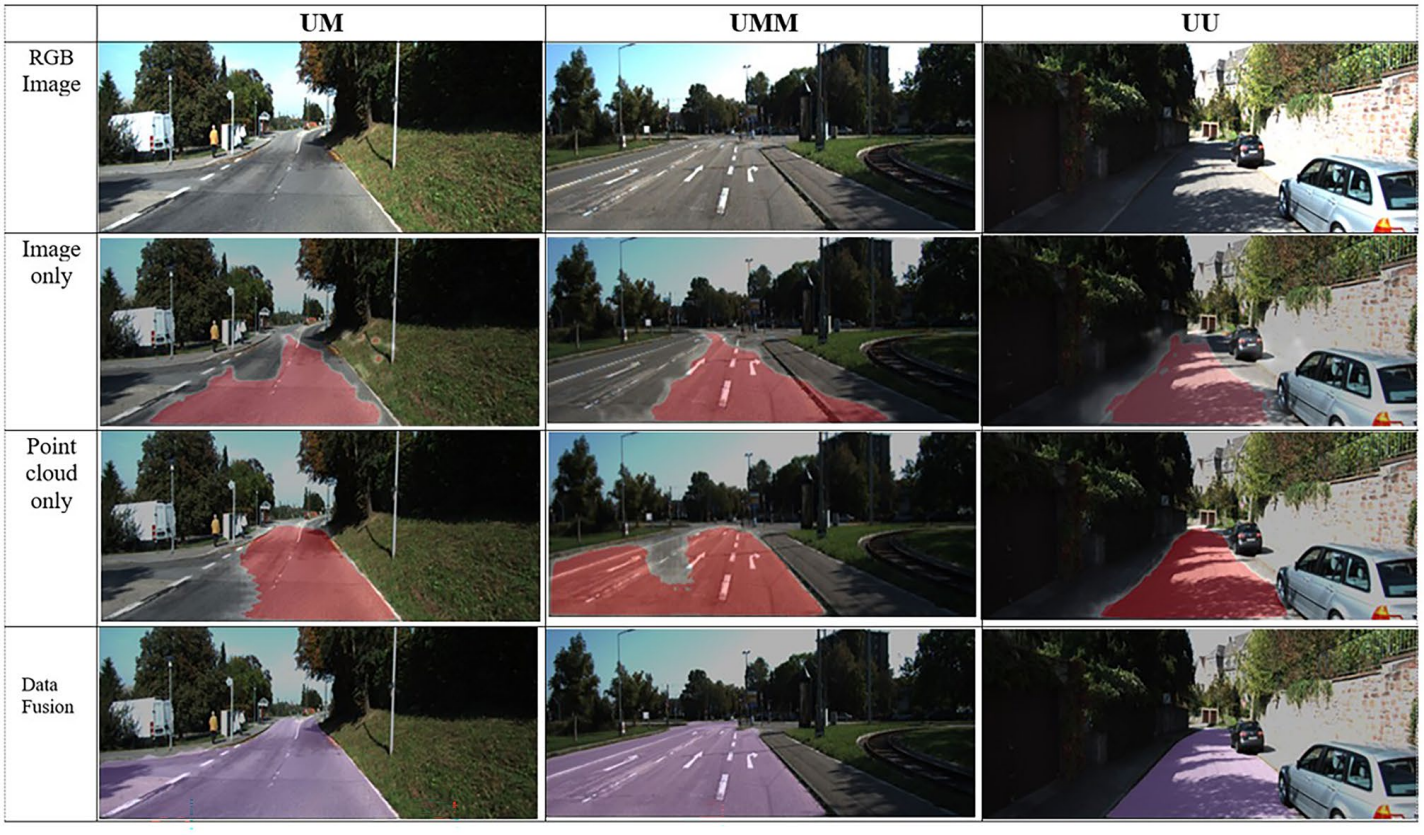
Visual results. The first row to the last row are the original images and the results of the image-based, point cloud-based, and proposed fusion methods, respectively.

In addition, we fetch some examples of the road segmentation results on the validation dataset in Fig. 6. Each line presents an image from the UM, UMM and UU datasets. Obviously, in the case of image only, when there are many shadows on the road (second row in Fig. 6), the road cannot be observed accurately. The point cloud modality, on the other hand, is less affected by illumination; hence, it gives better output than the RGB image modality, as illustrated in the third row of Fig. 6. However, in the case of point cloud only, it does not easily detect roads accurately if the height difference from the roadside is small. In the last row in Fig. 6, some misclassified regions in both separate modalities are corrected after fusion, and the performance can be enhanced with multimodality data fusion. The fusion method can effectively aggravate complementary features from the image and point cloud to achieve performance improvement for the single modality.

#### Evaluation on the KITTI benchmark test dataset

**Figure 7:**
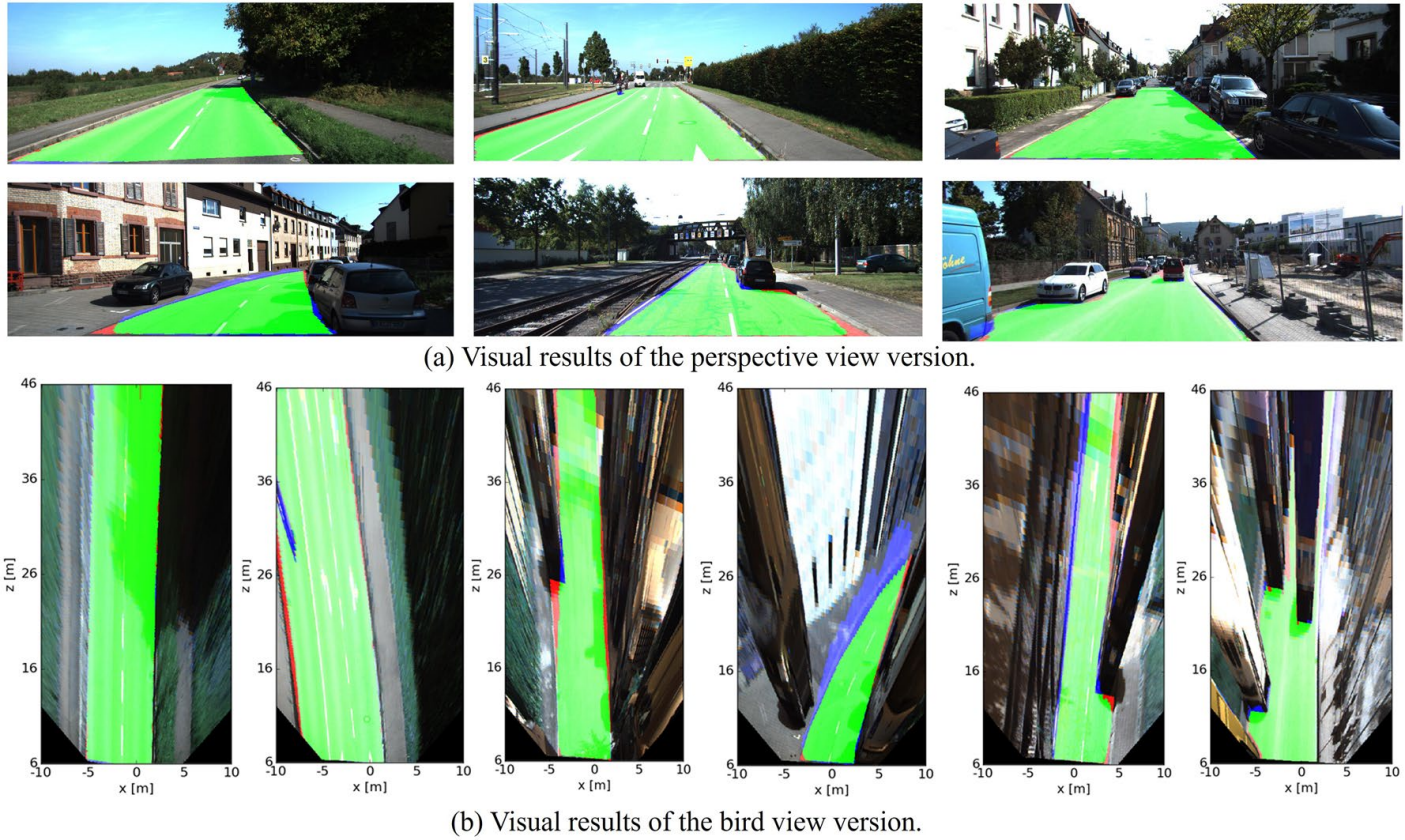
Visual results on the KITTI-ROAD test dataset.

As the KITTI road benchmark evaluates bird’s eye view results, the results in the perspective are mapped to a 400 $\times$ 800 bird’s eye view. Mapped images represent the accessibility of the region 40 m ahead (from 6 to 46 m) and 10 m on each side (or so), and then, they are submitted to the website for evaluation. Figure 7 shows some evaluation results.

Figure 7 illustrates some instances of road results yielded by the proposed method, with the perspective view of the image shown in Fig. 7a and a bird’s eye view shown in Fig. 7b. Each row of Fig. 7a matches the row in Fig. 7b. Before evaluation, the results of the perspective version are converted to the bird view version. Since the pixel resolution of the perspective decreases with the distance from the camera, distant pixels are more important when converting to the bird view. As seen in some areas, the edges of roads and shadows located by cars are slightly uneventful. Note that red indicates an incorrectly drivable region (false negatives), the blue area is the missing drivable region (false-positive), and green represents the correctly drivable region (true positives). This demonstrates that the proposed method has comparable performance.

**Figure 8:**
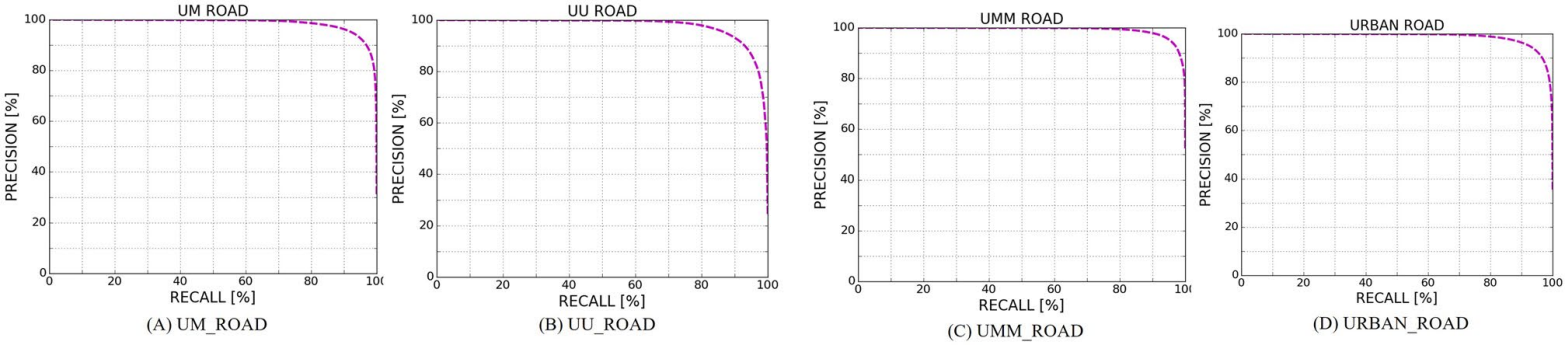
Precision–recall curves from the evaluation server.

Table 2 shows the statistical test results of 4 scenarios obtained directly from the evaluation server on the UMM_ROAD dataset. The main indicator, MaxF, reaches 95.41%, and the average MaxF on the entire test set reaches 94.27%. The UU scenario has the lowest performance compared to other scenarios because of its multiple complex environments and because it is more irregular than the other datasets. Furthermore, Fig. 8 shows the precision–recall results on the testing set for each urban scenario.

**Table 2:** Performance of the proposed model on KITTI (BEV) (/%).

Modality	MaxF	AP	PRE	REC	FPR	FNR
UM_ROAD	94.54	93.23	94.57	94.52	2.47	5.48
UMM_ROAD	95.41	95.39	95.42	95.41	5.04	4.59
UU_ROAD	92.00	92.19	92.01	91.98	2.60	8.02
URBAN_ROAD	94.27	93.63	94.22	94.32	3.19	5.68

#### Comparison with other fusion-based methods

To prove effectiveness, we compare this method with several high-ranking methods leveraging multimodality data on the KITTI testing dataset, including deep learning-based algorithms (PLARD^11^ and LidCamNet^12^), feature-based algorithms (RES3D-Velo^14^), and CRF fusion-based methods (FusedCRF^18^, HybridCRF^19^ and MixedCRF^21^). The statistical performance comparison results on the UM, UMM, and UU subsets and the average results on all sets (URBAN_ROAD) are illustrated in Tables 3, 4, 5, 6.

**Table 3:** Comparison with Several Popular Fusion-based Methods on UM_ROAD (BEV) (/%).

Method	MaxF	AP	PRE	REC	FPR	FNR
PLARD	97.05	93.53	97.18	96.92	1.28	3.08
LidCamNet	95.62	93.54	95.77	95.48	1.92	4.52
RES3D-Velo	83.81	73.95	78.56	89.80	11.16	10.20
FusedCRF	89.55	80.00	84.87	94.78	7.70	5.22
HybridCRF	90.99	85.26	90.65	91.33	4.29	8.67
MixedCRF	91.57	84.68	90.02	93.19	4.71	6.81
Ours	94.54	93.23	94.57	94.52	2.47	5.48

**Table 4:** Comparison with Several Popular Fusion-based Methods on UMM_ROAD (BEV) (/%).

Method	MaxF	AP	PRE	REC	FPR	FNR
PLARD	97.77	95.64	97.75	97.79	2.48	2.21
LidCamNet	97.08	95.51	97.28	96.88	2.98	3.12
RES3D-Velo	90.60	85.38	85.96	95.78	17.20	4.22
FusedCRF	89.51	83.53	86.64	92.58	15.69	7.42
HybridCRF	91.95	86.44	94.01	89.98	6.30	10.02
MixedCRF	92.75	90.24	94.03	91..50	6.39	8.50
Ours	95.41	95.39	95.42	95.41	5.04	4.59

**Table 5:** Comparison with Several Popular Fusion-based Methods on UU_ROAD (BEV) (/%).

Method	MaxF	AP	PRE	REC	FPR	FNR
PLARD	95.95	95.25	96.25	95.65	1.21	4.35
LidCamNet	94.54	92.74	94.64	94.45	1.74	5.55
RES3D-Velo	83.63	72.58	77.38	90.97	8.67	9.03
FusedCRF	84.49	72.35	77.13	93.40	9.02	6.60
HybridCRF	88.53	80.79	86.41	90.76	4.65	9.24
MixedCRF	85.69	75.12	80.17	92.02	7.42	7.98
Ours	92.00	92.19	92.01	91.98	2.60	8.02

**Table 6:** Comparison with Several Popular Fusion-based Methods on URBAN_ROAD (BEV) (/%).

Method	MaxF	AP	PRE	REC	FPR	FNR
PLARD	97.03	94.03	97.19	96.88	1.54	3.12
LidCamNet	96.03	93.93	96.23	95.83	2.07	4.17
RES3D-Velo	86.58	78.34	82.63	90.92	10.53	9.08
FusedCRF	88.25	79.24	83.62	93.44	10.08	6.56
HybridCRF	90.81	86.01	91.05	90.57	4.90	9.43
MixedCRF	90.59	84.24	89.11	92.13	6.20	7.87
Ours	94.27	93.63	94.22	94.32	3.19	5.68

As Tables 3, 4, 5, 6 illustrate, our method acquires good results in four scenarios, which demonstrates that for different situations, this method is not only accurate but also robust. Furthermore, it is obvious that the method is competitive (third place). In particular, compared with deep learning-based approaches (PLARD^11^ and LidCamNet^12^), PLARD^11^ performs best, LidCamNet^12^ ranks second, and the MaxF values of our method rank third. The reason behind our method having a slightly lower performance than PLARD^11^ and LidCamNet^12^ is that the height map features are fused multiple times in the deep learning network in middle-level fusion.

Compared with these handcrafted CRF fusion approaches, our approach excels based on all criteria, and it performs best on the main index, MAF, in general, achieving 6.02%, 3.46%, and 3.68% improvements over the MAF values of FusedCRF^18^, HybridCRF^19^, and Mixed CRF^21^ on the URBAN_ROAD dataset, respectively. In general, our method has certain advantages: it can not only add the results (information) in the unary potential but also integrate the RGB image, the densified height and the depth images in pairwise potentials, which can increase the data density; the energy function is iteratively optimized by mean-field variational inference; and followed by such a probabilistic fusion process, the proposed model possesses a considerable error correction capability. All results are calculated on KITTI’s online evaluation server, and results from other studies are based on the values from KITTI’s website.

The time inference comparison is shown in Table 7, in which the proposed method ranks fourth among the methods listed. Each method uses different hardware, and there is no unified standard for real-time performance due to the different experimental configuration environments.

**Table 7:** Time inference comparison.

Method	Runtime (ms)	Environment
PLARD	160	GPU @ 2.5 Ghz (Python)
LidCamNet	150	GPU @ 2.5 Ghz (Python)
RES3D-Velo	360	1 core @2.5 Ghz (C/C++)
FusedCRF	2000	1 core @2.5 Ghz (C/C++)
HybridCRF	1500	1 core @2.5 Ghz (C/C++)
MixedCRF	6000	1 core @ 2.5 Ghz (Matlab+ C/CC++)
Ours	1200	1 core @ 2.5 Ghz (Python)

**Figure 9:**
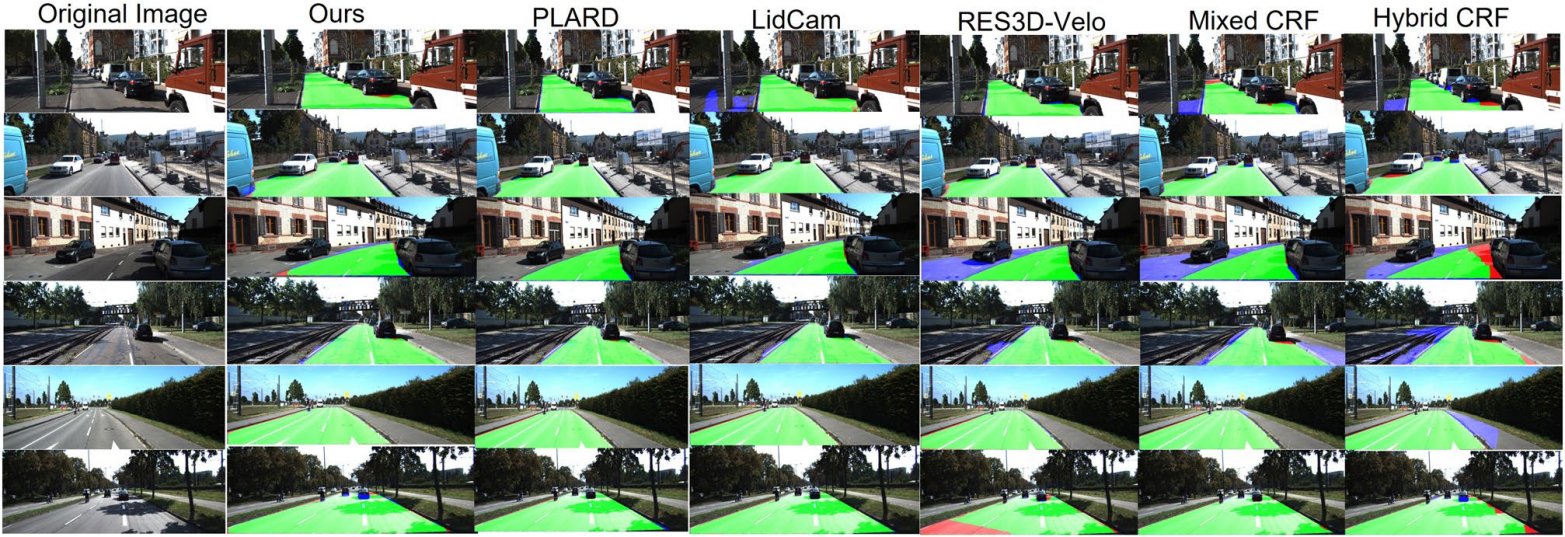
Visual results comparison on the KITTI-ROAD testing set.

Some distinctive results of the urban scenarios are also illustrated in Fig. 9. The first column is an RGB image; then, starting from the second column, road detection results from the methods mentioned in Table 6 are displayed. This model performs better than handcrafted CRF fusion-based methods in complex scenes.

## Conclusion

This paper proposes a camera–LiDAR sensor fusion method for road detection. It employs a novel R-CRF model to combine the results generated from the two sensors as the unary term. Densified LiDAR and RGB images are treated as pairwise terms in which edges are fully connected. Road detection is formulated as a two-class pixel labelling problem and iteratively optimized by mean-field variational inference. After the fusion process, the proposed model has great error correction ability. Extensive experiments are carried out on the KITTI dataset, and the results demonstrate that it performs better than single-modality-based methods. Compared with existing models, our method is competitive in detection accuracy.

## Data Availability

The datasets generated during the current study are available from the corresponding author on reasonable request.
